# Supplementary material for: Integrated Approach for the Optimization of the Sustainable Extraction of Polyphenols from a South American Abundant Edible Plant: Neltuma ruscifolia
Source: Foods. 2025 Aug 22;14(17):2927. doi: 10.3390/foods14172927 (PMC12427733; doi:10.3390/foods14172927)
Supplement: Supplementary file 1 [file foods-14-02927-s001.zip › foods-3810822-supplementary.pdf]

Integrated approach for the optimization of the sustainable extraction of polyphenols from a South American abundant edible plant: *Neltuma ruscifolia*

Giuliana S. Seling[1,2], Roy C. Rivero[1,3], Camila Sisi[1], Verónica M. Busch[1,3]* and M. Pilar Buera[2,4]*

Supplementary files

<u>Optimization Factors and their respective levels</u>

To determine the optimal extraction conditions for each method, the factors were kept within the studied ranges, and a multiple-response optimization criterion was applied. During optimization, each response was maximized and weighted according to its functional relevance: antioxidant capacity was assigned the highest importance (5), total phenolic content (TPC) an importance of 4, and total flavonoids (TF) an importance of 3. This weighting scheme prioritized obtaining an optimal extract for each method with the highest antioxidant capacity, associated primarily with elevated phenolic content and, secondarily, flavonoid content.

Table S1: Response Surface Experimental Designs for the three extraction methods. Design factors: Time in minutes (t); Temperature in °C (T); Ethanol content % (ET); Amplitude % (A) and Heating Intensity in watts (HI). Experimental data for response parameters: Antioxidant capacity in mmol Trolox equivalents/100 mL (DPPH); Total flavonoids (TF) and Total polyphenolic content (TPC).

	Dynamic maceration-assisted extraction						Ultrasound-assisted extraction						Microwave-assisted extraction						
	Factors			Responses			Factors			Responses			Factors				Responses		
	t	T	Et	DPPH	TF	TPC	t	A	ET	DPPH	TF	TPC	t	T	Et	NI	DPPH	TF	TPC
**1**	25	20	100	0.15±0.01	0.85±0.27	22.2±0.7	1	20	50	0.17±0.01	0.69±0.04	33.7±3.6	10	50	50	480	0.25±0.03	4.97±0.07	44.5±1.1
**2**	10	20	50	0.36±0.01	6.13±0.23	56.4±1.3	1	100	50	0.15±0.01	1.18±0.13	31.3±1.1	25	35	100	160	0.20±0.01	0.75±0.10	14.2±0.4
**3**	25	35	50	0.38±0.01	5.80±0.63	50.4±0.6	8	100	0	0.15±0.02	2.98±0.07	34.2±1.8	25	50	50	800	0.35±0.01	4.77±0.27	51.8±0.3
**4**	40	35	100	0.17±0.01	0.91±0.01	20.8±1.2	1	60	0	0.25±0.02	4.26±3.07	28.5±0.1	25	35	50	480	0.29±0.01	4.01±0.17	44.6±0.2
**5**	25	80	100	0.18±0.01	0.98±0.20	22.4±1.0	15	100	50	0.35±0.01	3.81±0.30	46.0±0.9	25	35	50	480	0.32±0.01	4.80±0.03	50.0±1.3
**6**	10	35	0	0.22±0.04	1.75±0.10	37.0±0.3	8	60	50	0.19±0.02	5.40±0.10	37.3±0.1	10	35	50	160	0.29±0.02	4.44±0.13	45.9±1.2
**7**	10	50	50	0.36±0.01	4.97±0.07	52.3±2.6	8	20	100	0.18±0.01	0.88±0.17	8.0±0.4	0	35	50	800	0.27±0.01	3.67±0.23	42.4±2.5
**8**	40	20	50	0.33±0.01	4.21±0.10	44.7±0.8	8	60	50	0.20±0.01	0.91±0.07	38.6±0.5	25	35	0	800	0.16±0.01	0.68±0.03	27.5±1.2
**9**	25	20	0	0.23±0.02	1.15±0.03	35.1±0.3	8	20	0	0.15±0.01	2.04±0.27	42.6±0.2	0	20	50	480	0.25±0.02	3.01±0.03	41.8±0.2
**10**	25	50	0	0.27±0.01	3.67±0.43	43.9±2.2	15	60	100	0.06±0.01	1.41±0.03	10.3±0.7	25	50	0	480	0.20±0.01	0.88±0.10	40.0±1.2
**11**	25	35	50	0.37±0.01	5.50±0.60	28.8±0.5	15	60	0	0.18±0.01	0.68±0.03	43.4±1.1	25	20	50	160	0.23±0.01	3.41±0.37	38.1±0.4
**12**	2	35	50	0.37±0.01	4.54±0.76	48.9±0.7	15	20	50	0.27±0.01	2.04±0.07	49.4±0.1	25	35	0	160	0.13±0.01	1.71±0.07	27.2±0.1
**13**	40	50	50	0.49±0.01	6.03±0.20	62.9±0.2	1	60	100	0.07±0.01	1.11±0.07	6.6±0.7	25	50	100	480	0.22±0.01	0.82±0.37	20.4±0.8
**14**	10	35	100	0.15±0.01	7.60±0.17	11.9±0.4	8	60	50	0.22±0.01	4.01±0.10	40.0±3.0	10	35	50	800	0.27±0.01	4.14±0.17	42.0±0.7
**15**	40	35	0	0.26±0.01	1.55±0.03	36.8±1.5	8	100	100	0.06±0.01	1.35±0.03	10.1±0.5	0	35	0	480	0.31±0.01	1.08±0.17	33.2±0.4
**16**													40	35	50	160	0.29±0.02	3.97±0.20	44.5±1.1

**17**								25 50 50 160	0.39±0.01	4.70±0.20	51.2±1.5
**18**								25 35 50 480	0.30±0.01	3.87±0.10	46.6±3.6
**19**								10 20 50 480	0.19±0.01	2.14±0.23	38.1±3.3
**20**								25 35 90 800	0.05±0.01	0.52±0.07	16.4±0.3
**21**								40 50 50 480	0.35±0.01	4.07±0.23	49.4±2.3
**22**								25 20 90 480	0.02±0.01	0.81±0.01	8.1±0.5
**23**								25 20 0 480	0.12±0.02	0.38±0.07	29.1±0.5
**24**								40 35 90 480	0.05±0.01	0.28±0.03	14.2±1.2
**25**								10 35 90 480	0.26±0.02	0.38±0.07	12.5±0.2
**26**								25 20 50 800	0.03±0.01	0.89±0.25	42.9±4.1
**27**								10 35 0 480	0.18±0.01	0.28±0.03	31.9±0.5
Model type	Quad.	Quad.	Quad.	Model type	Quad.	Quad.	Quad.	Model type	Quad.	Quad.	Quad.
Model Significance (p-value)	<0.0001	<0.0001	<0.0001	Model Significance (p-value)	<0.0001	0.1301	<0.0001	Model Significance (p-value)	<0.0001	<0.0001	<0.0001

R2	0.9664	0.9338	0.9855	R2	0.8452	0.5539	0.9770	R2	0.9196	0.9657	0.9551
R2 adj	0.9512	0.9006	0.9787	R2 adj	0.7378	0.2671	0.9548	R2 adj	0.8865	0.9506	0.9386
Lack of Fit	0.0638	0.9209	0.6456	Lack of Fit	0.3680	----	0.1537	Lack of Fit	0.2289	0.0583	0.0729
Adeq. Precision	26.84	15.87	39.22	Adeq. Precision	11.37	----	24.60	Adeq. Precision	19.56	22.79	23.27

Table S2: Fitted quadratic models for design factors: Time in minutes (t); Temperature in °C (T); Ethanol content % (ET); Amplitude % (A) and Heating Intensity in watts (HI). Experimental data for response parameters: Antioxidant capacity in mmol Trolox equivalents/100 mL (DPPH); Total flavonoids (TF) and Total polyphenolic compounds (TPC).

Treatment		Fitted quadratic models
DME	I.	$\mathbf{DPPH}=3.59E^{-1}+1.75E^{-2}\cdot t+3.03E^{-2}\cdot T+4.62E^{-2}\cdot ET+3.60E^{-2}\cdot t\cdot T-1.82E^{-2}\cdot t\cdot ET-2.10E^{-3}\cdot T\cdot ET+1.48E^{-2}t^2+9.00E^{-3}T^2-1.62E^{-1}ET^2$
	II.	$\mathbf{TF}=4.93+3.46E^{-1}\cdot t+5.63E^{-1}\cdot T-6.47E^{-1}\cdot ET+8.68E^{-1}\cdot t\cdot T+3.47E^{-1}\cdot t\cdot ET-3.91E^{-1}\cdot T\cdot ET^{-2}.39E^{-1}t^2+4.23E^{-3}T^2-3.81ET^2$
	III.	$\mathbf{CPT}=51.69+1.03\cdot t+3.23\cdot T-10.25\cdot ET+6.89\cdot t\cdot T+2.72\cdot t\cdot ET-6.25E^{-1}\cdot T\cdot ET-0.90E^{-1}t^2+3.15\cdot T^2-24.77\cdot ET^2$
UE	IV.	$\mathbf{DPPH}=2.11E^{-1}-5.73E^{-2}\cdot A-6.57E^{-2}\cdot ET-3.50E^{-3}\cdot t+2.42E^{-2}\cdot A\cdot ET+7.88E^{-2}\cdot A\cdot t+5.50E^{-3}\cdot ET\cdot t+6.95E^{-2}A^2-1.20E^{-1}ET^2+3.32E^{-2}t^2$
	V.	$\mathbf{TF}=2.99+3.994E^{-1}\cdot A-6.65E^{-1}\cdot ET+1.60\cdot t-3.32E^{-2}\cdot A\cdot ET-2.83E^{-1}\cdot A\cdot t+1.03\cdot ET\cdot t+6.32E^{-1}A^2-1.98ET^2-1.57t^2$
	VI.	$\mathbf{CPT}=39.91-3.42E^{-1}\cdot A-14.26\cdot ET+6.17\cdot t+2.28\cdot A\cdot ET-2.24E^{-1}\cdot A\cdot t-3.52\cdot ET\cdot t+1.26A^2-16.95ET^2+1.52E^{-1}t^2$
ME	VII.	$\mathbf{DPPH}=2.90E^{-1}+1.28E^{-2}\cdot t+5.32E^{-2}\cdot T-1.24E^{-2}\cdot NI-4.55E^{-2}\cdot ET+5.90E^{-3}\cdot t\cdot T+2.10E^{-3}\cdot t\cdot NI+1.27E^{-2}\cdot t\cdot ET-1.92E^{-2}\cdot T\cdot NI+1.40E^{-2}\cdot T\cdot ET-2.66E^{-2}\cdot NI\cdot ET-2.32E^{-2}t^2+1.00E^{-3}T^2+8.00E^{-3}NI^2-1.72E^{-3}ET^2$
	VIII.	$\mathbf{TF}=4.82+6.85E^{-2}\cdot t+5.65E^{-1}\cdot T-2.60E^{-1}\cdot NI-2.11E^{-1}\cdot ET-4.90E^{-1}\cdot t\cdot T-8.72E^{-2}\cdot t\cdot NI-2.81E^{-1}\cdot t\cdot ET+1.46E^{-1}\cdot T\cdot NI+1.11E^{-1}\cdot T\cdot ET+1.70E^{-1}\cdot NI\cdot ET-6.02E^{-1}t^2-6.91E^{-1}T^2-2.54E^{-1}NI^2-3.65ET^2$
	IX.	$\mathbf{CPT}=47.73+1.92\cdot t+4.61\cdot T-1.55E^{-2}\cdot NI-8.17E^{-1}\cdot ET+2.87E^{-1}\cdot t\cdot T+1.38\cdot t\cdot NI-1.36\cdot t\cdot ET-2.25E^{-1}\cdot T\cdot NI+8.38E^{-1}\cdot T\cdot ET+3.56E^{-1}\cdot NI\cdot ET-2.84t^2-4.05E^{-1}T^2-4.01E^{-1}NI^2-23.63ET^2$

Table S3. Extraction of *Neltuma spp.* (syn. *Prosopis* spp.) pods and bioactivity of the extract (highest values).

**Reference**	**Raw material**	**Extraction method**	**Results TPC/other**
**[49]**	*Neltuma pallida*	10% w/v hydroalcoholic extracts (Ethanol 96%, 70% and 45%) and two aqueous extracts (infusion and decoction)	90.65 mg GAE/g decoction
**[48]**	*N. alba (Prosopis alba) or N. chilensis*	Maceration hidroetanolic (without details)	TPC 6 mg GAE/g powder in N. alba, 7- 8 in N. chilensis.
**[50]**	*N. alba N. nigra*	10 g flour mixed with 100 mL of water or 96 ethanol. The mixture was homogenized for days at room temperature.	Total phenolics 4 mg GAE/1 g DW
**[32]**	*Prosopis spp.* from Tequisquiapan, Queretaro, Mexico	2 g sample mixed with 2.5 mL 80% ethanol (EtOH80) and thoroughly stirred for 2 min in a vortex	0.494 (mg GAE /g)
**[12]**	*Prosopis laevigata*	serial maceration with n-hexane, dichloromethane and methanol (MeOH) for 24 h	MeOH extract showed the most pronounced antimicrobial effect
**[51]**	*Prosopis chilensis*	MeOH under sonication (2 × 3 min each time), in 1:10 ratio, filtered and taken to dryness under reduced pressure	TPC 25 mg GAE/ g
**[33]**	*Prosopis laevigata*	Samples of 100 mg were extracted with one milliliter of40% ethanol in water (v/v) and centrifuged at 12,000 rpm/10 min.	TPC 8.87 mg GAE/g for mesocarp flour
**[34]**	*P. nigra and P. alba*	180 g flour was extracted three times with methanol: water (MeOH:H2O), 70:30 (1:10 w/v) with an ultrasonic bath for 1 h at 25°C.	2.1-6.7 mg GAE/g for P. alba and P. nigra

Response surface design for dynamic maceration extraction (DME)

Table S4. Analysis of Variance (ANOVA) of the Response Surface Model for Antioxidant Capacity in Dynamic Maceration Extraction.

Source	Sum of Squares	df	Mean Square	F-value	p-value
**Model**	0.2682	9	0.0298	63.84	< 0.0001
A-Time	0.0049	1	0.0049	10.52	0.0041
B-Temperature	0.0147	1	0.0147	31.55	< 0.0001
C-Ethanol %	0.0341	1	0.0341	73.09	< 0.0001
AB	0.0104	1	0.0104	22.26	0.0001
AC	0.0027	1	0.0027	5.68	0.0272
BC	0.0000	1	0.0000	0.0770	0.7842
A^2	0.0016	1	0.0016	3.47	0.0773
B^2	0.0006	1	0.0006	1.27	0.2735
C^2	0.1930	1	0.1930	413.48	< 0.0001
**Residual**	0.0093	20	0.0005		
Lack of Fit	0.0032	3	0.0011	2.93	0.0634
Pure Error	0.0062	17	0.0004		
**Cor Total**	0.2776	29			

Table S5. Analysis of Variance (ANOVA) of the Response Surface Model for total polyphenolic content in Dynamic Maceration Extraction

Source	Sum of Squares	df	Mean Square	F-value	p-value
**Model**	6882.56	9	764.73	143.96	< 0.0001
A-Time	17.07	1	17.07	3.21	0.0890
B-Temperature	166.84	1	166.84	31.41	< 0.0001
C-Ethanol %	1680.29	1	1680.29	316.31	< 0.0001
AB	380.01	1	380.01	71.54	< 0.0001
AC	59.15	1	59.15	11.13	0.0035
BC	3.12	1	3.12	0.5881	0.4526
A^2	5.64	1	5.64	1.06	0.3156
B^2	69.07	1	69.07	13.00	0.0019
C^2	4269.57	1	4269.57	803.74	< 0.0001
**Residual**	100.93	19	5.31		
Lack of Fit	9.68	3	3.23	0.5656	0.6456
Pure Error	91.25	16	5.70		
**Cor Total**	6983.49	28			

Table S6. Analysis of Variance (ANOVA) of the Response Surface Model for total flavonoids in Dynamic Maceration Extraction.

Source	Sum of Squares	df	Mean Square	F-value	p-value
**Model**	107.22	9	11.91	28.19	< 0.0001
A-Time	1.28	1	1.28	3.02	0.0994
B-Temperature	5.07	1	5.07	12.00	0.0028

C-Ethanol %	4.47	1	4.47	10.57	0.0044
AB	6.03	1	6.03	14.27	0.0014
AC	0.4814	1	0.4814	1.14	0.3000
BC	1.22	1	1.22	2.89	0.1065
A^2	0.3434	1	0.3434	0.8125	0.3793
B^2	1.08	1	1.08	2.55	0.1280
C^2	87.00	1	87.00	205.87	< 0.0001
**Residual**	7.61	18	0.4226		
Lack of Fit	0.0780	2	0.0390	0.0829	0.9209
Pure Error	7.53	16	0.4706		
**Cor Total**	114.83	27			

Response surface design for ultrasound-assisted extraction (UE)

Table S7. Analysis of Variance (ANOVA) of the Response Surface Model for Antioxidant Capacity in Ultrasound-assisted Extraction.

Source	Sum of Squares	df	Mean Square	F-value	p-value
**Model**	0.1862	9	0.0207	21.73	< 0.0001
A-Amplitude	0.0302	1	0.0302	31.73	< 0.0001
B-Ethanol %	0.0624	1	0.0624	65.60	< 0.0001
C-Time	0.0001	1	0.0001	0.1374	0.7155
AB	0.0039	1	0.0039	4.06	0.0600
AC	0.0242	1	0.0242	25.44	< 0.0001
BC	0.0002	1	0.0002	0.2566	0.6189
A^2	0.0266	1	0.0266	27.97	< 0.0001
B^2	0.0844	1	0.0844	88.70	< 0.0001
C^2	0.0065	1	0.0065	6.88	0.0178
**Residual**	0.0162	17	0.0010		
Lack of Fit	0.0008	2	0.0004	0.3693	0.6973
Pure Error	0.0154	15	0.0010		
**Cor Total**	0.2023	26			

Table S8. Analysis of Variance (ANOVA) of the Response Surface Model for total polyphenolic content in Ultrasound-assisted Extraction.

Source	Sum of Squares	df	Mean Square	F-value	p-value
**Model**	5636.06	9	626.23	80.08	< 0.0001
A-Amplitude	1.70	1	1.70	0.2172	0.6471
B-Ethanol %	2711.47	1	2711.47	346.74	< 0.0001
C-Time	452.81	1	452.81	57.91	< 0.0001
AB	41.69	1	41.69	5.33	0.0338
AC	0.3346	1	0.3346	0.0428	0.8386
BC	70.58	1	70.58	9.03	0.0080
A^2	10.31	1	10.31	1.32	0.2668
B^2	1861.92	1	1861.92	238.10	< 0.0001
C^2	0.1514	1	0.1514	0.0194	0.8910
**Residual**	132.94	17	7.82		

Lack of Fit	40.51	3	13.50	2.05	0.1537
Pure Error	92.42	14	6.60		
**Cor Total**	5769.00	26			

Table S9. Analysis of Variance (ANOVA) of the Response Surface Model for total flavonoids in Ultrasound-assisted Extraction.

Source	Sum of Squares	df	Mean Square	F-value	p-value
**Model**	27.70	9	3.08	1.93	0.1301
A-Amplitude	1.27	1	1.27	0.7987	0.3866
B-Ethanol %	3.53	1	3.53	2.22	0.1585
C-Time	6.86	1	6.86	4.30	0.0569
AB	0.0088	1	0.0088	0.0055	0.9417
AC	0.2128	1	0.2128	0.1336	0.7202
BC	2.83	1	2.83	1.78	0.2038
A^2	2.01	1	2.01	1.26	0.2797
B^2	19.76	1	19.76	12.40	0.0034
C^2	5.51	1	5.51	3.46	0.0842
**Pure Error**	22.31	14	1.59		
**Cor Total**	50.00	23			

Response surface design for microwave-assisted extraction (ME)

Table S10. Analysis of Variance (ANOVA) of the Response Surface Model for Antioxidant Capacity in microwave-assisted extraction.

Source	Sum of Squares	df	Mean Square	F-value	p-value
**Model**	0.4732	14	0.0338	27.77	< 0.0001
A-Time	0.0034	1	0.0034	2.76	0.1061
B-Temperature	0.0642	1	0.0642	52.71	< 0.0001
C- Heating intensity	0.0033	1	0.0033	2.72	0.1084
D-Ethanol %	0.0451	1	0.0451	37.08	< 0.0001
AB	0.0003	1	0.0003	0.2304	0.6343
AC	0.0000	1	0.0000	0.0242	0.8773
AD	0.0009	1	0.0009	0.7354	0.3971
BC	0.0025	1	0.0025	2.05	0.1617
BD	0.0016	1	0.0016	1.28	0.2654
CD	0.0056	1	0.0056	4.63	0.0385
A^2	0.0048	1	0.0048	3.96	0.0547
B^2	8.876E-06	1	8.876E-06	0.0073	0.9325
C^2	0.0006	1	0.0006	0.4827	0.4919
D^2	0.2710	1	0.2710	222.63	< 0.0001
**Residual**	0.0414	34	0.0012		
Lack of Fit	0.0154	10	0.0015	1.42	0.2289
Pure Error	0.0260	24	0.0011		
**Cor Total**	0.5146	48			

Table S11. Analysis of Variance (ANOVA) of the Response Surface Model for total polyphenolic content in microwave-assisted extraction.

Source	Sum of Squares	df	Mean Square	F-value	p-value
**Model**	9170.11	14	655.01	57.75	< 0.0001
A-Time	88.29	1	88.29	7.78	0.0082
B-Temperature	511.06	1	511.06	45.06	< 0.0001
C- Heating intensity	0.0058	1	0.0058	0.0005	0.9821
D-Ethanol %	1601.04	1	1601.04	141.15	< 0.0001
AB	0.6573	1	0.6573	0.0579	0.8111
AC	15.27	1	15.27	1.35	0.2532
AD	14.77	1	14.77	1.30	0.2609
BC	0.4038	1	0.4038	0.0356	0.8514
BD	5.63	1	5.63	0.4968	0.4852
CD	1.02	1	1.02	0.0896	0.7664
A^2	78.82	1	78.82	6.95	0.0121
B^2	1.61	1	1.61	0.1417	0.7087
C^2	1.58	1	1.58	0.1390	0.7113
D^2	5467.64	1	5467.64	482.05	< 0.0001
**Residual**	431.01	38	11.34		
Lack of Fit	179.44	10	17.94	2.00	0.0729
Pure Error	251.58	28	8.98		
**Cor Total**	9601.13	52			

Table S12. Analysis of Variance (ANOVA) of the Response Surface Model for total flavonoids in microwave-assisted extraction.

Source	Sum of Squares	df	Mean Square	F-value	p-value
**Model**	148.51	14	10.61	64.28	< 0.0001
A-Time	0.0956	1	0.0956	0.5794	0.4521
B-Temperature	5.51	1	5.51	33.38	< 0.0001
C- Heating intensity	1.20	1	1.20	7.24	0.0112
D-Ethanol %	1.00	1	1.00	6.08	0.0192
A-Time	1.63	1	1.63	9.88	0.0036
AC	0.0424	1	0.0424	0.2568	0.6158
AD	0.6321	1	0.6321	3.83	0.0591
BC	0.1038	1	0.1038	0.6288	0.4336
BD	0.0837	1	0.0837	0.5073	0.4815
CD	0.2321	1	0.2321	1.41	0.2444
A^2	3.16	1	3.16	19.17	0.0001
B^2	4.06	1	4.06	24.59	< 0.0001
C^2	0.5406	1	0.5406	3.28	0.0797
D^2	122.62	1	122.62	743.01	< 0.0001
**Residual**	5.28	32	0.1650		
Lack of Fit	2.46	9	0.2734	2.23	0.0583
Pure Error	2.82	23	0.1226		
**Cor Total**	153.79	46			

Optimized *Neltuma ruscifolia* extract by ultrasound-assisted extraction: HPLC chromatogram

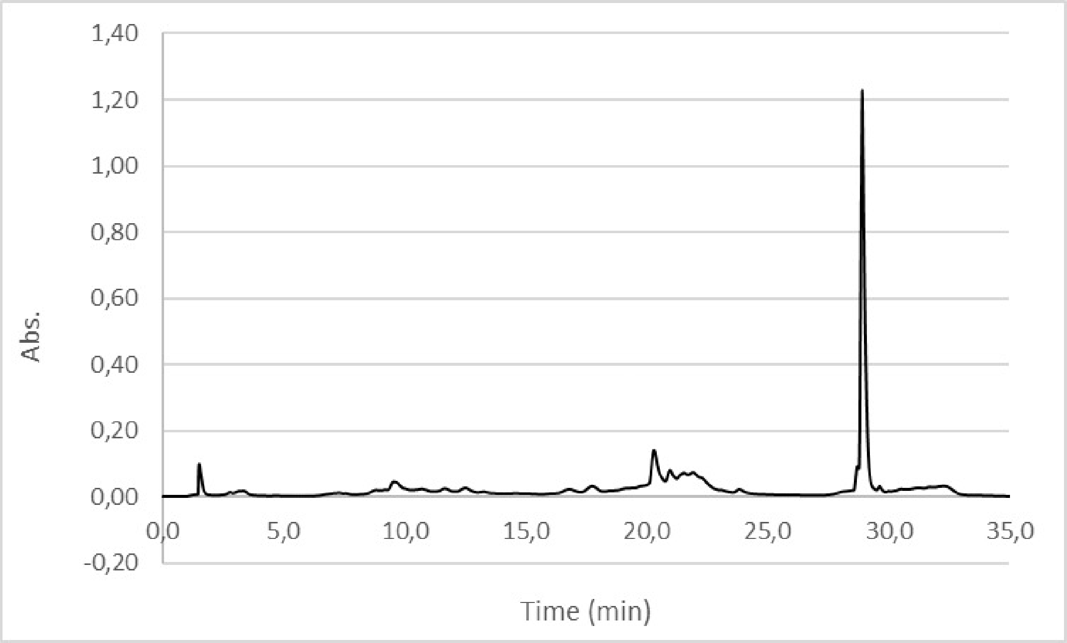

Figure S1. HPLC chromatogram of the optimized *Neltuma ruscifolia* extract obtained by ultrasound-assisted extraction. The chromatogram was recorded at 271 nm to maximize peak visualization, while quantification of each identified compound was carried out at its specific wavelength of maximum sensitivity, according to the nature of the compound.
